# Effects of Polyphenol Consumption on Recovery in Team Sport Athletes of Both Sexes: A Systematic Review

**DOI:** 10.3390/nu14194085

**Published:** 2022-10-01

**Authors:** Mariana Sánchez Díaz, Adrián Martín-Castellanos, Valentín E. Fernández-Elías, Olga López Torres, Jorge Lorenzo Calvo

**Affiliations:** 1 Departamento de Deportes, Facultad de Ciencias de la Actividad Física y el Deporte, Universidad Europea de Madrid, 28670 Madrid, Spain; 2 Departamento de Deportes, Facultad de Ciencias de la Actividad Física y el Deporte, Universidad Politécnica de Madrid, 28040 Madrid, Spain

**Keywords:** intermittent sports, ergogenic aids, sports nutrition, muscle damage

## Abstract

Previous studies have shown that polyphenol consumption enhances recovery of the muscle after exercise-induced muscle damage (EIMD). However, EIMD markers have not been studied by sport type. The main aim of this research was to perform a systematic review to determine the efficacy of polyphenolic consumption in increasing muscle recovery for performing team sport skills. Eligible studies included, following PICOS structure, presented at least one of the following outcomes: maximal isometric voluntary contraction (MVIC); countermovement jump (CMJ); delayed onset muscle soreness (DOMS); 20 m sprint test; creatine kinase (CK); and C-reactive protein (hsCRP). A structured search was carried out following the Preferred Reporting Items for Systematic Review and Meta-Analyses (PRISMA) guidelines. The risk of bias was assessed using the PEDro scale tool. The review showed a possibly positive impact of polyphenol consumption on recovery after EIMD in team sports athletes. No differences were found between sexes. Considering the limitations, there is moderate to very low certainty of polyphenol supplementation effects on recovery of team sport females and males. A dose of 60 mL/day, divided into two times per day, ingested for >7 days may present positive effects on muscle function and muscle soreness in team sport athletes. However, further investigation is required, specifically in females.

## 1. Introduction

Team sports are characterized by intermittent bouts of high-intensity exercise such as jumping, sprinting and tackling that require implementation of complex sport-specific skills and cognitive tasks over a prolonged period [1,2]. These types of sports (such as soccer, rugby and volleyball) performance depend on both aerobic and anaerobic capacity, requiring a high volume of energy turnover and eccentric high-intensity exercise muscle actions resulting in exercise-induced muscle damage (EIMD) and therefore decreasing physical performance [1,2,3]. On the other hand, oxidative stress is an imbalance between prooxidant (free radicals) production and the body’s antioxidant defense system [4]. Free radicals are oxygen-containing molecules with one or more unpaired electrons that allow them to easily interact with other species. Research indicates that oxidative stress is evident following muscle-damaging exercise and seems to have a potent role in the adaptation process following EIMD [5].

According to Harty et al. [6], EIMD is a transient phenomenon caused by unaccustomed, damaging exercise. It is characterized by structural damage to myofibers and secondary inflammation resulting from leukocyte infiltration into the damaged tissues. Signs and symptoms appear immediately after and often persist for several days and up to 14 days after exercising [6,7]. EIMD has a direct impact on athletes’ functional and exercise capacity, muscle soreness, disturbing sense of force production and limb position, decreased pressure pain threshold (PPT) and elevated levels of intramuscular enzymes such as creatine kinase (CK), lactate dehydrogenase (LDH) and myoglobin (MYO) [6,7]. Additionally, EIMD often results in elevations in markers of inflammation such as C-reactive protein (CRP) and various interleukins [6]. Symptoms are associated with losses in muscle function and increases in muscle soreness, impairing athletes’ exercise performance (7).

The focus of many sports’ nutrition strategies is to maximize the recovery from exercise and prepare for the next exercise burst. Numerous nutrients and functional foods have been examined for their potential to decrease oxidative stress and therefore EIMD [7]. Polyphenols, by their antioxidant and anti-inflammatory properties, may reduce oxidative stress markers, inflammation markers and EIMD, thereby enabling an earlier return to normal muscle strength/force [7]. Somerville et al. [8] evaluated the overall effect of polyphenols on human performance and suggested that its supplementation leads to a moderate improvement in performance with no reported adverse effects; moreover, this effect may be detected in any athlete regardless of the type of sport or its duration.

Polyphenols are mainly classified by chemical structure and distinguished from other chemical compounds by the combination of one or more hydroxyl compounds with aromatic rings (phenols). These molecules can be subclassified into stilbenes, lignans, phenolic acids and flavonoids [9,10]. Phenolic acids and flavonoids are the most common in the human diet, with main sources including fruits, vegetables, fruit juices, tea, coffee, red wine, cereals and chocolate [11].

Numerous studies have investigated the efficacy of foods high in polyphenols. One polyphenolic-rich food commonly used for investigation is tart cherry (TC) juice. Vitale et al. [12] summarized data from 11 studies using TC supplementation in athletes to accelerate recovery, for example, after eccentric elbow flexion contractions [12,13]. Other polyphenolic-rich foods commonly used are pomegranate juice [14], New Zealand blueberry [15], beetroot juice [16], cocoa pods [17] and black currant nectar [18]. However, there is inconsistency in the results of the different studies and the different methodologies may be the reason for these differences [17].

Different systematic reviews and meta-analyses have examined the effects of fruit supplements on indices of muscle damage and physical performance measures following different EIMD protocols [3,19,20]. After consuming polyphenol-rich foods, juices and concentrates, 24, 48 and 72 h post-EIMD are the most commonly studied times for comparing muscle damage, inflammatory and oxidative stress markers between a placebo group and a supplementation group [19,20]. For MVIC, CMJ and DOMS, maximal benefits of polyphenols supplementation for accelerating recovery seem to occur at 48- and 72-h post-exercise [20]. However, Carey et al. [3] demonstrated the ability of polyphenols to enhance recovery of skeletal muscle strength and soreness four days post-EIMD, but specifically with polyphenol treatments that contain flavonoids.

Goncalves et al. [21] reviewed 320 articles to summarize the effects of dietary phenolics on physical performance and recovery in athletes and suggested that the impact of phenolic supplementation is dependent on dose, nutritional habits, type of polyphenolics consumed, redox status and training status of the individuals and the type of training. Other authors have studied the supplementation of polyphenols in different health-related variables (cardiovascular diseases, blood pressure, lipid biomarkers, or platelet aggregation) [22]. One experimental study analyzed the differences between sexes; however, conclusions showed a lack of evidence in showing whether male and female athletes have the same performance response after polyphenol intake [23]. Moreover, to our knowledge, until today, no systematic review and meta-analysis has synthesized evidence on polyphenols and recovery after EIMD exclusively in team sports athletes and, more specifically, in female athletes. The objective of this systematic review and meta-analysis is to assess the effects of consuming polyphenols on physical performance markers of muscle damage/soreness (MVIC, CMJ, DOMS), blood markers of muscle damage (CK, CRP) and oxidative stress markers (TBARS, TAC), looking for specific differences between sexes.

## 2. Materials and Methods

This systematic review is presented following the Preferred Reporting Items for Systematic Review and Meta-Analyses (PRISMA 2020). The study was assessed and registered on the International Prospective Register of Systematic Reviews (PROSPERO) (CRD42021284599, 6 November 2021) [24].

### 2.1. Eligibility Criteria

For eligibility (inclusion/exclusion) criteria the PICOS model was applied [25]: P (Population): “healthy young male and female team field sport athletes, excluding individual sports”; I (Intervention): “all polyphenol interventions were considered if they were protocolized in conjunction with a training plan (the doses were systematically provided to patients according to a predefined strategy)”; C (comparison): “studies comparing an intervention or experimental group with a placebo group with similar characteristics”; O (outcome): “a trial had to include physical performance tests after EIMD, muscle damage biomarkers and/or oxidative stress biomarkers”, S (study): randomized control trials studies with a parallel or a cross-over design published in English”.

### 2.2. Search Strategy

Studies that investigated the effects of polyphenols on recovery were identified by conducting a structured individual search in the following databases from inception to August 2021: Scopus, PubMed, Web of Science (WOS), SportDiscus and Cochrane. Specifically, the Scopus search equation was: ((“intermittent sports” OR “team sport” OR “team sports” OR “soccer” OR “football” OR “field hockey” OR “lacrosse” OR “basketball” OR “volleyball” OR “American football” OR “rugby” OR “baseball” OR “softball” OR “cricket”) AND (“polyphenol” OR “polyphenols”) AND (“muscle damage” OR “muscle pain” OR “muscle soreness”)).

### 2.3. Study Selection

The search for published studies was performed by one of the authors (MSD) and disagreements were resolved through discussions between two of the authors (MSD and JLC). The eligibility of the studies was performed in a standardized manner. All duplicates were removed, as well as all other articles that were not clinical trials. Titles and abstracts were screened and any relevant one that appeared to help this study was included. Studies that satisfied the inclusion criteria were selected and the full text was reviewed.

### 2.4. Data Extraction

After applying the eligibility criteria, the following information was extracted from each included study: source of the article (authors and year of publication), the study design (parallel or crossover, blinded subjects, sample size), the characteristics of the population (age, gender, sport, level) and the polyphenol supplementation used in each study (kind, dose, duration, content).

Intending to perform a Meta-analysis with the Review Manager program [26], the authors extracted data of means and standard deviations (SD) for selected outcomes (MVIC, CMJ, DOMS, 20 m sprint, CK and CRP). Due to lack of precise variable data and the scarcity of eligible studies to be included in the Meta-analysis, finally this could not be performed. Risk of bias was examined using the Physiotherapy Evidence Database Scale (PEDro) [27].

## 3. Results

### 3.1. Study Selection

**Figure 1:**
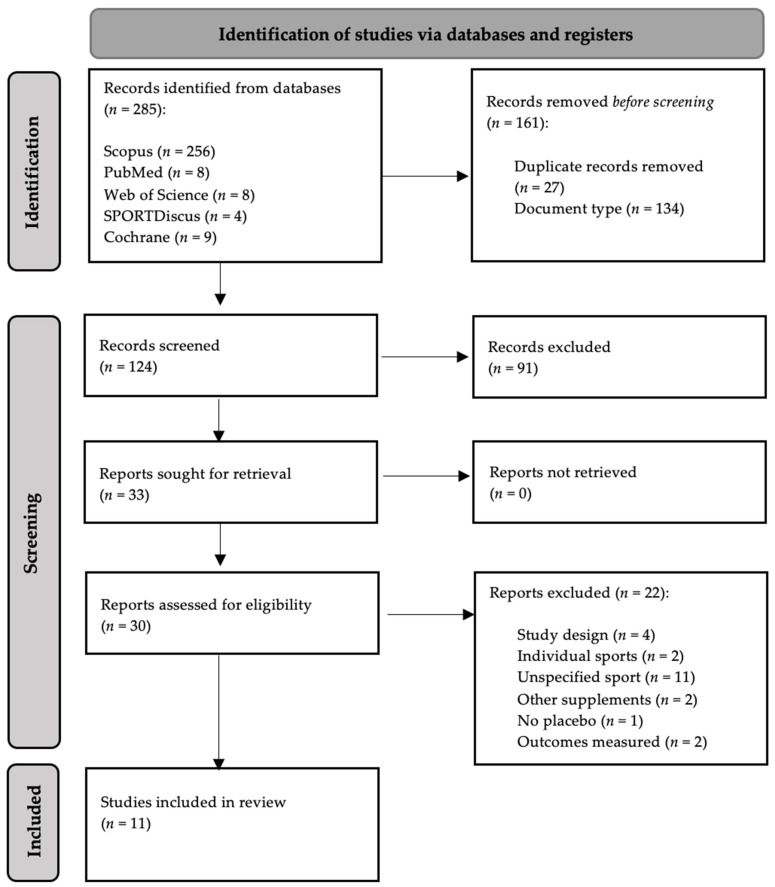
Selection of studies according to an adapted version of PRISMA 2020 flow diagram [24].

A total of 11 studies were identified for inclusion in this review. Ten out of the 11 were conducted exclusively in men and one had a mixed population. The search of Scopus, PubMed, Web of Science (WOS) and SportDiscus identified 285 records. Of these, 124 were screened after removing 27 for duplication and 134 for the type of study (systematic reviews and book chapters). After reviewing the titles and the abstracts 91 were removed because they did not meet the inclusion criteria. A total of 30 studies were examined and 22 were excluded (see flow diagram for reasons, Figure 1).

### 3.2. Study Characteristics

#### 3.2.1. Methods

Of the 11 studies included in the review seven had a parallel design and four employed a cross-over design (see Table 1). Participants were randomly positioned in either a placebo group or an experimental group in all the selected studies. In eight of them, both investigators and participants were blind to the treatment allocation and in three studies investigators were aware of the treatment allocation.

#### 3.2.2. Participants

A total of 209 participants were included. 141 of them were collegiate or semi-professional team sport athletes and 68 were elite or professional team sport athletes. Only one of the studies had a mixed sample and included female participants (12 women and 8 men), although sex comparation analysis were not conducted. The age range of the studies included in the review was around 15–30 years.

#### 3.2.3. Intervention

As mentioned above, all studies compared a placebo group with an experimental one that consumed a polyphenol-rich concentrate or supplements such as the following: green tea extract (GT) [28,29], Montmorency tart cherry concentrate (MC) [2,34], beetroot juice (BTJ) [16], jamelon nectar [30], tart cherry juice (TCJ) [32,33], grape juice (GJ) [35] and chokeberry juice [36].

### 3.3. Outcomes

#### 3.3.1. Physical Performance

Of the 11 studies included in the systematic review three measured MVIC [2,16,33], five CMJ [2,16,32,33,34], four sprint speed (20 or 30 m) [2,30,33,36] and five used DOMS as a marker of muscle soreness [2,31,32,33,34] (see Table 1).

#### 3.3.2. Muscle Damage Biomarkers

Of the 11 studies, seven evaluated CK [2,16,28,29,30,33,35], three evaluated CRP [2,16,33], two evaluated LDH [29,30] and one evaluated AST [29] (see Table 1). 

#### 3.3.3. Oxidative Stress Biomarkers

Seven out of the 11 studies evaluated oxidative stress biomarkers. Three evaluated thio-barbituric acid–reacting substances (TBARS) [28,35,36]; four evaluated total antioxidant status (TAS) [28] or total antioxidant capacity (TAC) [29,30,36]; and two evaluated lipid hydroperoxides (LOOH) [2,16] (see Table 1). 

### 3.4. Risk of Bias

The quality analysis (Physiotherapy Evidence Database Scale (PEDro) checklist) yielded the following results (see Table 2): (a) The quality scores ranged from 6 to 10; (b) The average score was 8.7; (c) Of the 11 included studies, five were categorized as “very high quality” (10 points), three were categorized as “high quality” (8–9 points) and three were categorized as “medium quality” (6–7 points). The highest scores were in the following items: no. 2 (random allocation), no. 5 (blinded subjects), no. 10 (between-group statistical comparisons) and no. 11 (points estimates and variability). On the other hand, the lowest scores were in items: no. 1 (eligibility criteria) and no. 3 (concealed allocation). It is important to consider that no. 1 criterion is not used to calculate the PEDro score (Table 2. PEDro scale [27]).

### 3.5. Synthesis of Results

#### 3.5.1. Physical Performance Tests Findings in This Review

Table 1 shows that in two [2,33] out of the three [2,16,33] studies that evaluated MVIC, results were significantly higher compared to the placebo group. CMJ was significantly higher in the experimental group compared to placebo in three [2,16,33] of the five [2,16,32,33,34] studies that evaluated that variable. Of the four studies that evaluated sprint speed (20 or 30 m) [2,30,33,36], two [2,33] had a significantly positive effect on the experimental group. DOMS and three [31,32,34] of the five [2,31,32,33,34] who included the variable had no significant differences. 

No sex differences were analyzed in any physical performance variables (CMJ, MVIC and 20 m sprint) measured in the study that included female athletes. However, in the same study, all the physical performance tests (see Table 1) had a significantly positive result in the experimental group compared to the placebo [33]

#### 3.5.2. Effect of Polyphenols on Muscle Damage Biomarkers

Seven of the studies evaluated CK [2,16,28,29,30,33,35] and none of them found significant differences when comparing an experimental group with a placebo. Moreover, three evaluated CRP [2,16,33] and none showed significant results. One evaluated AST [29], two LDH [29,30], and none showed significance at any level.

#### 3.5.3. Oxidative Stress Biomarkers Findings in This Review

Only one [35] of the three [28,35,36] studies that evaluated TBARS found a significant decrease compared to placebo. The study that evaluated TAS [2] was the only one that found significant differences and the other three studies [29,30,36] that evaluated TAC instead of TAS found no changes in the results compared to placebo. Polyphenol consumption had no effects on LOOH [2,16] (see Table 1).

## 4. Discussion

To the best of the authors’ knowledge, this is the first systematic review to investigate the effects of polyphenolic supplement ingestion after EIMD in team sports players. The review of the 11 studies shows that the consumption of polyphenols has a statistically significant positive impact on physical performance tests and a positive but not statistically significant effect on muscle damage biomarkers after EIMD in team sports players (see Table 1). However, evidence, in general, is moderate to very low and inconsistent, especially for oxidative stress biomarkers.

Tart cherry is one of the most commonly used polyphenolic-rich food supplements for its anti-inflammatory and antioxidant capacity [12]. Vitale et al. [12] summarized data from 11 studies using TC. They suggested that its supplementation in athletes in a state where the priority is recovery and not adaptation may be beneficial, especially during excessive inflammatory/oxidative stress during single-day intense training/competition or multiday tournaments [12]. In this review, five [2,31,32,33,34] of the 11 studies used a dose of 30 mL twice a day of TC supplementation. However, methodological differences between studies were seen in the number of days and the type of EIMD applied. A 3-day TC supplementation before and after (12–60 h post) a 90 min soccer match showed no group differences for muscle damage biomarkers (CMJ, RSI, MS) and subjective wellbeing [32]. Twice-daily consumption of TC before and after an 80 min RU match did not attenuate MS or alter wellbeing in the following 3 days [31]. Supplementation of 60 mL/day of MC in rugby players for 7 days showed no effects of TC on markers of muscle soreness and function [34]. In addition, Bell et al. [2] evaluated the same dose of TC during 8-day supplementation and in semi-professional male soccer players after completing an adapted LIST version. Results showed no group differences in hsCRP, LOOH and CK. Nevertheless, at 48 h, the TC group sprint time was significantly lower; the experimental group also had higher physical performance indices of CMJ, MVIC and 5-0-5 agility, specifically 72 h post-exercise; moreover, DOMS was lower compared to the PLA group [2]. The results mentioned before contrast with Bell et al. [37] where MVIC in trained cyclists did not change at 72 h post-exercise. The findings of Bell et al. [2] are consistent with Quinlan et al. [33]. They demonstrated that TC in the same dose (2 × 30 mL/day during 8 days) in different team sports players (soccer, hockey and netball) accelerates recovery (*p* < 0.05) at 24 and 48 h post-LIST in a 20-m sprint, CMJ and MVIC, without differences throughout recovery for DOMS, CRP and CK [33]. Morgan et al. [17] also found improvements in recovery of CMJ up to 48 h post-exercise; however, they used cocoa as a polyphenolic-rich supplement. 

The above, in terms of timing, is consistent with Doma et al.’s [19] systematic review and meta-analysis; they showed that MVIC was significantly greater for the supplementation condition than placebo at 24 and 48 h post-EIMD. Another systematic review and meta-analysis by Rickards et al. [20] studied the effects of consuming polyphenol after EIMD using MVIC and CMJ as primary markers of muscle damage and DOMS as a primary marker of muscle soreness. They observed that the maximal benefits for accelerating recovery of muscle function while reducing muscle soreness after consuming polyphenol-rich foods, juices and concentrates occurred at 48 and 72 h post-exercise [20]. Moreover, Carey et al. [3] demonstrated the ability of polyphenols to enhance recovery of skeletal muscle strength and soreness four days post-EIMD, but specifically with polyphenol treatments that contain flavonoids.

It might be interesting to take into account the origin of the supplement. Considering that the kind or storage time or form (raw, frozen, dehydrated, cooked, etc.) of the food can vary the polyphenol concentration, studies must report that matter. All articles in this review reported the kind (green tea extract, Montmorency tart cherry concentrate, beetroot juice, etc.), dose, timing and form of the polyphenol supplementation (see Table 1), but none reported the storage time. Moreover, it is necessary to know the concentration of the specific type of polyphenol according to the chemical structure subclassification (stilbenes, lignans, phenolic acids, or flavonoids) [9,10]. It is necessary to report the concentration to make a more accurate comparison of the relationship between dose and effect. In addition, comparing the automated oxygen radical absorbance capacity (ORAC) of the different supplements used in the included articles would help make a much more realistic comparison [38]. 

Clifford et al. [16] tested the effects of BTJ using a 250 mL dose twice a day for 3 days. They evaluated the impact of polyphenols on recovery between 2 RST with players of different team sports. After muscle damage, CMJ height appeared to recover quicker in BTJ vs. PLA (*p* = 0.048). The maintenance of RI performance was also significantly greater in the experimental group (*p* = 0.030). However, for MVIC, CK, hs-CRP, PC and other oxidative stress biomarkers, there were no group or interaction effects [16]. In addition, BTJ for 3 days after a marathon race does not attenuate muscle soreness, enhance the recovery of muscle function or attenuate biochemical markers of inflammation and muscle damage in marathon runners [39].

One of the problems that caught the authors’ attention was the remarkably low participation of females in this review. Sex-specific differences might exist in the effects of polyphenols on performance and recovery in intermittent sports. Nevertheless, as already mentioned, studies performed on female athletes in team sports are scarce, as well as those comparing and analyzing the differences between sexes. In this regard, in a meta-analysis by Rickards et al. [20], a relation of 4:1 was observed with only female participation. In another review conducted by Di Lorenzo et al. [22], 37 studies were included, only four were performed in a female-only population and three out of the four did not obtain significant differences between the group that took the polyphenols and the control group. On the other hand, in the same review, nine of the 37 studies analyzed were conducted in males and four obtained positive effects on different variables when consuming polyphenols [22]. Only one study examined the differences between sexes, having positive results [22]. On the contrary, a study performed with runners showed no sex differences when consuming beetroot juice in enhancing performance [23]. In this review, Quinlan et al. [33] is the only one of the 11 selected studies that included female team sport athletes; however, no methodological analysis took gender into account throughout the study.

Few sex differences have been seen in different parameters and variables related to the topic. Some evidence suggests that 8-day TC supplementation in an exclusively female population may be a practical nutritional intervention to help attenuate the symptoms of muscle damage and improve recovery (especially on CMJ) [40]. Studies differentiating sex or exclusively in females are few to none even though a higher consumption of polyphenol-rich food products by the female population has been demonstrated and it is known that the ability/impact of polyphenol supplements may be affected by the intake of dietary polyphenols [20,41]. Moreover, it is important to consider the menstrual cycle and that the female hormone estrogen has a possible impact on reducing the inflammatory response of the muscle. Therefore, further investigation is required to determine specific recommendations according to gender [42,43].

### 4.1. Limitations

The main limitation of this systematic review was the scarcity of studies on polyphenolic supplementation in team sports players, resulting in a lack of data for the selected variables. As mentioned before, the authors intended to present a meta-analysis. However, they ran into limitations of a lack of available raw data in the published articles, followed by difficulty contacting some of the authors to ask for the missing information. Another limitation was related to the protocols used in the different studies resulting in an inability to compare variables (e.g., not presenting the mean and standard deviation of its results, or different pain scales applied for DOMS). The authors developed a forest plot that included two variables (CK and CRP) from two studies [2,16]; however, due to the scarcity of data followed by high heterogeneity, the authors decided to exclude the Meta-analysis from this study. For those reasons, the results of this systematic review must be interpreted carefully. Furthermore, findings demonstrate the need for more well-designed studies with clear variables that can be interpreted together and, of course, more female samples.

### 4.2. Future Research

To understand better the effects of polyphenolic supplement ingestion after EIMD in team sports players, further investigation is required considering the following recommendations: (1) selection of athletes according to the type of sport; (2) application of a general protocol of polyphenol supplementation, including the specific kind and concentration of polyphenol supplement used following the laboratory analysis data (to avoid the risk of relying on claims made on the label or by the manufacturer); (3) inclusion of more female athletes.

## 5. Conclusions

Results of this systematic review have shown moderate to very low certainty that polyphenol supplementation improves performance and muscle function and reduces muscle soreness and oxidative stress biomarkers of team sport athletes after EIMD, especially when rapid recovery is required.

A dose of 60 mL/day during 7 days of polyphenolic supplementation appears to have a positive effect in accelerating recovery following prolonged, repeat sprint activity, such as soccer and rugby. However, further investigation is required to clarify the duration and dose.

Polyphenol supplementation does not appear to reduce creatine kinase (CK) and C-reactive protein (CRP) at 0, 1, 24 and 72 h post EIMD in team sport athletes. However, further investigation is required to clarify the effects of polyphenols on muscle damage biomarkers.

## Figures and Tables

**Table 1:** RCT design characteristics for all studies included in this review.

Study Id		Population	Exposure to Polyphenols		Exercise	Outcomes Analyzed		
Author/s-Year	Study Design	Characteristics (Sample Size) Age	Kind Dose, Timing	Polyphenolic Content	EIMD	Physical Performance Test	Muscle Damage Biomarkers	Oxidative Stress Biomarkers
Jówko et al., 2012 [28]	RCT Double-blind Parallel	Local club soccer players (*n* = 16) 22.4 ± 3.4	Green tea polyphenols (GTP) 640 mg per 1 day	* 1 capsule: Total of 320 mg polyphenols, including about 250 mg catechins	Muscle-endurance test	n/a	Pre, 5 min and 24 h post: ↔ CK	Pre, 5 min and 24 h post: ↔ TBARS ↔ UA ↔ Total catechins ↔ TAS ↔ SOD
Bell et al., 2016 [2]	RCT Double-blind Parallel	Semi-professional male soccer players (*n* = 16) 25 ± 4 years	Montmorency tart cherry concentrate (MC) 2 × 30 mL per day for 8 days	* 1000 mL: Total flavonoids: 73.5 mg cyanidin-3-glucoside Total phenols: 178.8 mg of GAE	12 × 20 m sprint followed by LIST (6 × 15 min sections).	24, 48, 72 h post: ↑ MVIC ↑ CMJ ↓ DOMS (VAS) ↑ 20 m sprint ↑ 5-0-5 Agility	0, 1, 3, 5, 24, 48, 72 h post: ↔ CK ↔ hsCRP	0, 1, 3, 5, 24, 48, 72 h post: ↔ LOOH
Clifford et al., 2016 [16]	RCT Double-blind Parallel	Collegiate male team sports players (*n* = 20) (Soccer (*n* = 10), rugby (*n* = 5), basketball (*n* = 2) hockey (*n* = 2) or handball (*n* = 1)) 21–23 years	Beetroot juice (BTJ) 2 × 250 mL per day for 3 days	-	2 RST: RST1 (20 × 30 m) and RST2 (72 h later).	Pre, post, 24, 48 and 72 h after RST1 and post and 24 h after RST2: ↔ MVIC ↑ CMJ ↑ RI ↔ PPT	Pre, post, 2.5, 24, 48 and 72 h after RST1 and post, 2.5 and 24 h after RST2: ↔ CK ↔ hsCRP	Pre, post, 2.5, 24, 48 and 72 h after RST1 and post, 2.5 and 24 h after RST2: ↔ PC ↔ LOOH ↔ A•−
Hadi et al., 2016 [29]	RCT Double-blind Parallel	University male soccer players (*n* = 49) 18–25 years	Green tea extract (GTE) Sour tea extract (STE) 450 mg per day for 6 weeks	-	No specifications given	n/a	Pre and post (6 weeks): ↔ CK ↔ AST ↔ LDH	Pre and post (6 weeks): ↑ TAC (STE)
Assunção et al., 2018 [30]	RCT Double-blind Parallel	Elite high school male handball players (*n* = 25) 18 ± 2.4 years	*Syzygium cumini* (SC)/ jamelon nectar 10 mL per kilogram per day for 28 days	-	4 w of periodization of medium-intensity endurance training, maximal power and speed, sport-specific strength and power and techno-tactical skills.	Pre and post (4 weeks): ↔ Vertical jump height ↔ 20 m shuttle run test ↔ Running anaerobic sprint test (RAST)	Pre and post (4 weeks): ↔ CK ↔ LDH	Pre and post (4 weeks): ↔ TAC
Kupusarevic et al., 2019 [31]	RCT Double-blind Crossover	Rugby union (RU) elite male players (*n* = 10) 28 ± 4 years	Tart cherry juice (TCJ) 2 × 30 mL per day for 5 days (2 days before, the day of the match and 2 days after).	-	RU 80 min match	24, 48, 72 h post: ↔ DOMS ↔ Subjective wellbeing	n/a	n/a
Abbott et al., 2020 [32]	RCT Double-blind Crossover	Professional male soccer players (*n* = 10) 19 ± 1 years	Tart cherry juice (TCJ) 2 × 30 mL per day per 3 days	-	90-min soccer match	12, 36, 60 h post: ↔ CMJ ↔ DOMS ↔ RSI ↔ Subjective wellbeing	n/a	n/a
Quinlan et al., 2019 [33]	RCT Single-blind Parallel	Team sports male (*n* = 8) and female (*n* = 12) players (Soccer, hockey, or netball) 26 ± 4 years	Tart cherry juice (TCJ) 2 × 30 mL per day for 8 days	-	LIST (6 × 15 min sections) followed by 12 × 20 m sprint.	Pre and 1, 24, 48 h post: ↑ MVIC ↑ Agility ↑ CMJ ↓ DOMS ↑ 20 m sprint	Pre and 1, 24, 48 h post: ↔CK ↔ CRP	n/a
Morehen et al., 2021 [34]	RCT Single-blind Crossover	Professional Rugby male players (*n* = 11) 18 ± 1 years	Montmorency cherry juice (MC) 2 × 30 mL per day for 7 days (5 days before and 2 after the match)	* 30 mL: 320 mg of anthocyanins	RU match	24 pre, 24 and 48 h post: ↔ CMJ ↔ DOMS	n/a	n/a
Martins et al., 2020 [35]	RCT Double-blind Crossover	National competitors’ male volleyball players (*n* = 12) 16 ± 0.6 years	Grape juice (GJ) 400 mL per day for 14 days	* 2.08 ± 0.02 g EAG/L Total flavonoids: 0.258 ± 0.00 g EQ/L	3 volleyball match simulations	Pre and post each match: ↔ Vertical jump height ↔ Handgrip strength (HG)	Pre and post each match: ↔ CK	Pre and post each match: ↓ TBARS ↔ Carbonyls ↓ DNA damage
Stankiewicz et al., 2021 [36]	RCT Double-blind Parallel	Semi-professional male soccer players (*n* = 20) 15.8 ± 0.7 years	Chokeberry juice 2 × 100 mL per day for 7 weeks	* 165.3 mg/100 mL of anthocyanins	Regular physical training program (microcycle) during the 7 w of supplementation. “The beep test”: Pre and post the 7-w supplementation period, maximal multistage 20-m shuttle run test	Before and after 7 weeks: ↔ 20 m sprint	n/a	0, 3, 24 h post the beep test. ↔ TAC ↔ TBARS ↔ 8-OHdG

* content stated in the study; - content not stated; n/a not applicable; ↔ no significant difference; ↑ significantly higher from the placebo group; ↓ significantly lower from the placebo group; abbreviations: RCT, randomized controlled trials; CK, creatine kinase; TBARS, thio-barbituric acid; UA, uric acid; TAS, total antioxidant status; SOD, superoxide dismutase; GAE, gallic acid equivalent; LIST, Loughborough intermittent shuttle test; MVIC, maximal isometric voluntary contraction; CMJ, countermovement jump; DOMS, delayed onset muscle soreness; hsCRP, high-sensitivity C-reactive protein; LOOH, lipid hydro-peroxides; RST, repeated sprint test; RI, reactive strength index; PPT, pressure-pain threshold; PC, protein carbonyls; A-, ascorbyl free radical; AST, aspartate aminotransferase; LDH, lactate dehydrogenase; TAC, total antioxidant capacity.

**Table 2:** Risk of bias graph: Review of authors’ judgment on each risk of bias item from PEDro scale presented as percentages across all included studies.

	Jówko et al., 2012 [28]	Bell et al., 2016 [2]	Clifford et al., 2016 [16]	Hadi et al., 2016 [29]	Assunção et al., 2018 [30]	Kupusarevic et al., 2019 [31]	Abbott et al., 2020 [32]	Quinlan et al., 2019 [33]	Morehen et al., 2021 [34]	Martins et al., 2020 [35]	Stankiewicz et al., 2021 [36]	Studies Meeting Criterion *n* (%)
1.Eligibility criteria	●	●	●	●	●	●	●	●	●	●	●	6 (55%)
2.Randomized allocation	●	●	●	●	●	●	●	●	●	●	●	11 (100%)
3.Concealed allocation	●	●	●	●	●	●	●	●	●	●	●	7 64%)
4.Comparable at baseline	●	●	●	●	●	●	●	●	●	●	●	9 (82%)
5.Blinded subjects	●	●	●	●	●	●	●	●	●	●	●	11 (100%)
6.Blinded therapists	●	●	●	●	●	●	●	●	●	●	●	9 (82%)
7.Blinded assessors	●	●	●	●	●	●	●	●	●	●	●	9 (82%)
8.Adequate follow-up	●	●	●	●	●	●	●	●	●	●	●	9 (82%)
9.Intention to treat analysis	●	●	●	●	●	●	●	●	●	●	●	9 (82%)
10.Between-group comparisons	●	●	●	●	●	●	●	●	●	●	●	11 (100%)
11.Point estimates and variability	●	●	●	●	●	●	●	●	●	●	●	11 (100%)
Total points	10 (100%)	6 (60%)	9 (90%)	10 (100%)	10 (100%)	9 (90%)	10 (100%)	7 (70%)	8 (80%)	10 (100%)	7 (70%)	

Note: ● indicates low risk of bias, ● indicates high risk of bias.

## Data Availability

Data sharing not applicable.
